# Psychometric Properties of the Chinese Wayfinding Effectiveness Scale

**DOI:** 10.1093/geroni/igaa057.884

**Published:** 2020-12-16

**Authors:** Yi-Chen Chiu, Donna Algase

**Affiliations:** 1 Chang Gung University, Taoyuan County, Taiwan (Republic of China); 2 University of Michigan, Ann Arbor, United States

## Abstract

Being unable to find one’s way is a terrifying experience accompanied by feelings of fear or frustration. Persons with cognitive impairment (PWCIs) are apt to get lost and show difficulty in wayfinding effectiveness. Therefore, it is important to develop an instrument to measure the wayfinding effectiveness in PWCIs in northern Taiwan. This was a cross-sectional design with 180 PWCIs to examine the psychometric properties of the Chinese Wayfinding Efficient Scale (CWE) developed by Algase. An exploratory Factor Analysis with varimax rotation was computed to explore the underline conceptual structure of the CWE. The results derived 6 factors: FDPFE = Finding a destination and a path in familiar environments; FDPUFE=Finding a destination and a path in unfamiliar environments; SDAS = Sense of direction and analytic strategies; SDGS = Sense of direction and global strategies; LMD = Landmark and distance in familiar/unfamiliar environments; MAP = Using a map in familiar/unfamiliar environments. This solution explained 66.50% of variance. The internal consistency values for the total and subscales were excellent. Item 25 was single loaded, therefore, deleted. One-week test-retest reliability of the CWE total scale and subscales were estimated using a subset of data (n = 15) by calculating ICC. One subscale, Finding a distance and a path in familiar environments (FDPFE), did not have significant test-retest reliability but the rest of the subscales and total scale had excellent test-retest reliability (.81-1.00). The initial psychometric properties of the CWE were acceptable. Further research should explore the possible associate factors.

